# Diffuse Muscular Pain, Skin Tightening, and Nodular Regenerative Hyperplasia Revealing Paraneoplastic Amyopathic Dermatomyositis due to Testicular Cancer

**DOI:** 10.1155/2012/534236

**Published:** 2012-12-17

**Authors:** Sarah Norrenberg, Valérie Gangji, Véronique Del Marmol, Muhammad S. Soyfoo

**Affiliations:** ^1^Department of Dermatology, Hôpital Erasme, Université Libre de Bruxelles, 1070 Bruxelles, Belgium; ^2^Department of Rheumatology and Medical Physicine, Hôpital Erasme, Université Libre de Bruxelles, 808 Route de Lennik, 1070 Bruxelles, Belgium

## Abstract

Paraneoplastic dermatomyositis (DM) associated with testicular cancer is extremely rare. We report the case of a patient with skin tightening, polymyalgia, hypereosinophilia, and nodular regenerative hyperplasia revealing seminoma and associated paraneoplastic DM.

## 1. Introduction

Dermatomyositis (DM) encompasses a heterogeneous group of multisystemic inflammatory myopathies with variable clinical and laboratory characteristics, affecting mainly the skin and proximal skeletal musculature [1, 2]. The prevalence of DM is between 0.5 and 1 case per 100.000 people [1]. The criteria of Bohan and Peter have been reviewed and newly adapted by A. A. Amato in 2003. This new classification for the idiopathic inflammatory myopathies was approved by the MSG and ENMC workshop [2]. The main subgroups described are the primary idiopathic DM in adults, paraneoplastic DM, amyopathic DM, juvenile DM, overlap syndromes, drug-induced DM, primary idiopathic PM, “inclusion body” myositis, eosinophilic myositis/perimyositis, and orbital myositis with giant cell myocarditis [1, 2]. Principal clinical features include symmetrical proximal muscular weakness, Gottron's papules, Gottron's sign, heliotrope eruption, skin erythema (V-sign erythema, shawl sign), photosensitive poikiloderma, and periungual erythema/telangiectasia [1, 2]. Diagnosis of DM can be made on the basis of biopsy-confirmed classical skin findings, proximal muscle weakness, and elevated muscle enzymes (creatinin Phosphokinase, CPK; Aldolase). Electromyography and/or muscle biopsy can also be performed to confirm active myositis. Amyopathic DM is distinguished from classic DM by the absence of clinically evident myopathy. Furthermore, Stonecipher et al. [3] have defined 3 subgroups of ADM: (1) no subjective or objective evidence of myopathy, (2) no subjective muscle weakness but abnormalities detected by objective tests, (3) subjective muscle weakness but no objective evidence of myopathy.

The relative risk of neoplasia is increased the first year after diagnosis of DM and is about 26/1.000.000 [4]. Variable frequencies of malignancies with DM have been reported in the literature, from 6% to 60% [4–6], around 15% and 34% in Western countries [7]. Dermatomyositis and cancer usually arise within one to five years of one another [8]. The most reported malignancies are nasopharyngeal carcinoma (predominantly in Chinese population), ovarian carcinoma, lung carcinoma, pancreatic carcinoma, lymphomas, breast carcinoma, liver carcinoma, colorectal carcinoma, and gastric carcinoma [3, 5]. The risk of developing a specific type of neoplasia with DM is variable following different populations [4, 6, 8]. Patients with older age at onset of disease (>45 years) and male gender are more likely to develop malignant diseases [5, 7].

We hereby report the case of a patient with fibromyalgia-like syndrome and testicular cancer revealing dermatomyositis.

## 2. Case Report

**Figure 1:**
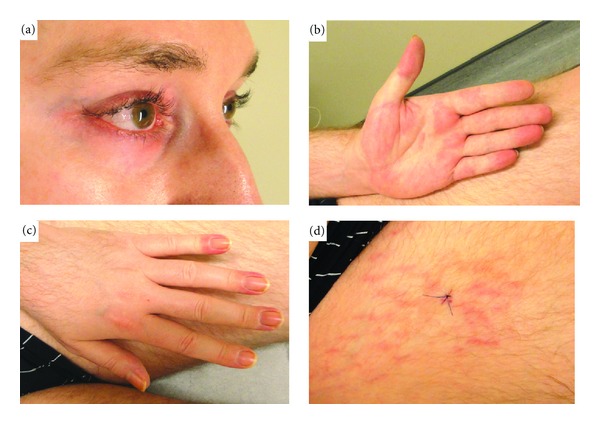
Cutaneous features of DM. (a) Heliotrope erythema, (b) palmar erythema, (c) periungual erythema, and (d) flagellate erythema.

**Figure 2:**
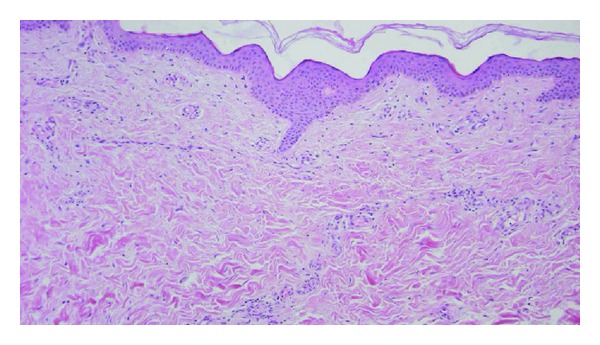
The cutaneous biopsy of flagellate erythema (magnification ×10) depicting atrophic epiderma. In the superficial derma, there was a slight inflammatory infiltrate, essentially composed of mononuclear cells and rare telangiectasies.

A 30-year-old man of Caucasian origin was seen in an outpatient rheumatology clinic for diffuse polyarticular and muscular pain. The patient described important morning stiffness of more than 2 hours, the sensation of skin rigidity, and diffuse myalgia predominating in the proximal muscles. His current medications include tetrazepam 50 mg/d, amitriptyline 75 mg/d (for depression), and oxycodone 80 mg/d. Initial physical examination showed normal vital parameters and absence of synovitis but revealed joint and muscle tenderness. Blood analysis showed normal levels of C-reactive protein and normal erythrocyte sedimentation rate, hypereosinophilia (700/mm^3^), and increased liver enzymes without increases in CPK levels. Electromyography (EMG) of the left thigh was performed but did not reveal any abnormalities. A liver biopsy was done and showed nodular regenerative hyperplasia (NRH) and no signs of malignancy. Serologies and other investigations were performed to rule out any parasitic infection as well as autoimmune diseases (Sjögren's syndrome and systemic sclerosis). Another EMG and a total body Magnetic Resonance Imaging were performed in 2010, but did not reveal any convincing element in favour of myopathy. Because of worsening physical condition with loss of weight, appetite, and skin tightening, an ^18^fluorodeoxyglucose PET-CT was performed and revealed a right testicular hypermetabolic mass, confirmed by ultrasonography. One month later, a large right orchidectomy was performed and histology revealed a pure seminoma of two centimeters, classified pT1NX. The patient condition worsened and he developed palmar erythema, amyotrophy, increased muscular weakness, heliotrope erythema, flagellate erythema on anterior thighs, palmar erythema, and sclerosis-like aspect of the skin (Figure 1). Capillaroscopy showed dermal microvasculitis characterized by tortuosities and diffuse edema. An ^18^fluorodeoxyglucose PET-CT scan was performed again and did not show any metastasis but revealed bilateral basal pulmonary infiltrates that were confirmed by a high-resolution computed chest tomography. Pulmonary function tests were performed and showed significantly reduced DLCO compatible with interstitial lung disease. A cutaneous biopsy of the flagellate erythema was done and histological analysis was compatible with a diagnosis of dermatomyositis (Figure 2). Treatment with Methylprednisolone 1 mg/kg daily improved the dermatological features and the liver tests but the muscular pain did not subside. AZA (2 mg/kg/d) was then added but the patient's condition still did not improve.

## 3. Discussion

We have reported the case of a patient presenting with testicular cancer, hypereosinophilia, and NRH unveiling the diagnosis of amyopathic DM. The diagnosis delay could be attributed to protean clinical and biological manifestations that misled the physicians in charge of the patient. For four years, the patient was believed to have a fibromyalgia-like syndrome because of the appealing symptoms of polymyalgia and myalgia concomitant with the paucity of clinical and biological signs of autoimmune involvement. Early in the disease, the patient presented with high levels of hepatic enzymes and hypereosinophilia, without elevation of CPK. NRH could explain the elevation of hepatic enzymes but the relationship with muscle weakness could not be made at that time. NRH has been described in association with collagen diseases [9], but to our knowledge, this is the first case associated with dermatomyositis. Hypereosinophilia could be linked with neoplasia [10] in the absence of patent evidence of parasitic infection.

Paraneoplastic DM associated with testicular cancer is extremely rare. In their review, Dourmishev et al. [11] reported 11 cases of DM associated with testicular cancer. One new case was also described by Tan et al. [12]. Patients were aged between 24 and 46 years old. There were six cases of nonseminomatous testicular cancer, two malignant teratoma, one intratubular germ-cell tumor, one mix testicular cancer with a pattern of seminoma, and three pure seminoma. Symptoms of DM appeared in seven cases before malignancy, three after orchiectomy and three after orchiectomy and chemotherapy. Half of the patients presented metastasis at diagnosis. All cases showed increased values of CPK, LDH, and liver enzymes that were associated with DM. Disappearance of the DM was totally different from case to case. Some resolved after treatment of cancer while others appeared after treatment as in the present case. Some patients responded to corticosteroids while others did not. Some cases required more effective immunosuppressants such as AZA, HCQ, or methotrexate. The extremely low incidence of paraneoplastic DM associated with seminoma is explained by the rarity of both diseases. The current incidence of testicular tumor is 63/100 000 per year [13]. The death rate is very low [13]. It is found in young male between 15 and 35 years old [12]. This explains the contrast with the age distribution in patients with paraneoplastic DM.

## 4. Conclusion

Paraneoplastic DM associated with testicular cancer is extremely rare. This is the first case of paraneoplastic amyopathic DM associated with testicular tumor. As in our case, other patients have developed DM after initial treatment of their neoplasia. No pathogenic mechanism underlying the triggering of DM following treatment of neoplasia has been suggested. This is probably due to the rarity of the phenomenon and the heterogeneous clinical presentation. Furthermore, DM could appear within years before or after diagnosis of malignancy. The disease can spontaneously disappear after treatment of the cancer or could be treated with initial high doses of MPN daily in a decreasing fashion [2]. First line treatment with MPN followed by adjunct of immunosuppressants seems to be the mainstay of treatment even if no randomized controlled trials are available.
